# The relationship of prescriber training level to restricted antibiotic prescribing appropriateness at a tertiary academic hospital: a retrospective study

**DOI:** 10.1017/ash.2025.10185

**Published:** 2025-10-19

**Authors:** Paul Greidanus, Ryan J. LeBlanc, Dima Kabbani, Stephanie W. Smith, Karen E. Doucette, Cecilia Lau, Serena Bains, Karen Fong, Jackson J. Stewart, Teagan Zeggil, Justin Z. Chen

**Affiliations:** 1 Core Internal Medicine, Department of Medicine, University of Alberta, Edmonton, AB, Canada; 2 Division of Infectious Diseases, Department of Medicine, University of Alberta, Edmonton, AB, Canada; 3 Pharmacy Services, Alberta Health Services, Edmonton, AB, Canada

## Abstract

**Objective:**

This study aimed to compare appropriateness of restricted antimicrobial prescriptions, as assessed by antimicrobial stewardship program (ASP) prospective audit and feedback (PAF), between those ordered by medical trainees versus staff. Secondary objectives were to determine whether certain timing factors and other independent variables impacted prescription appropriateness.

**Design:**

Single center, retrospective cohort study.

**Setting:**

The University of Alberta Hospital a 700-bed tertiary care hospital in Edmonton, Canada.

**Participants:**

Prescriptions of six health-authority restricted antibiotics subject to ASP PAF between 2018 and 2023. Cases were excluded if prescriber role or prescription dates or times were unavailable.

**Methods:**

Data from a local ASP quality improvement database was extracted. Multiple logistic regression analysis was completed with adjusted odds ratios (aOR) reported.

**Results:**

A total of 3,687 restricted antibiotic prescriptions subjected to PAF were included in this study, of which 1,163 (31.5%) were assessed as not appropriately prescribed. Prescriptions written by medical trainees did not have higher odds of appropriateness compared to staff (aOR 1.09 [95% CI 0.94–1.28], *P* = .25). Weekend prescriptions had a reduced odds of being appropriate (aOR 0.71 [0.60–0.84], *P* < .0001). Through the course of the Coronavirus Disease 2019 (COVID-19) pandemic, appropriateness improved from 56.2% (prepandemic), 71.5% (peri-pandemic) to 76.9% (postpandemic).

**Conclusions:**

No differences were noted in restricted antibiotic prescription appropriateness between medical trainees and staff. Weekend prescriptions were less likely to be appropriate. Improved appropriateness over time may be multifactorial, including implementation of ASP preceding the pandemic. Further studies examining timing factors associated with appropriateness are needed.

## Introduction

Antimicrobial resistance (AMR) is a growing problem and is associated with increased healthcare cost, longer hospital stays, and increased mortality.^1,2^ Antimicrobial stewardship programs (ASP) attempt to tackle AMR by developing coordinated strategies and interventions to target inappropriate antimicrobial use, especially broad-spectrum antimicrobials.^3,4^ Prospective audit and feedback (PAF) is a key stewardship strategy employed by ASPs.^5^ It is resource intensive especially if applied broadly to numerous antimicrobial targets, syndromes, and/or prescriber populations. ASPs are often faced with operational decisions to implement PAF selectively, such as to specific prescriber populations, given real-world resource limitations.

Alberta Health Services (AHS), the provincial health authority responsible for delivery of healthcare in the province of Alberta, employs formulary restriction to meropenem, imipenem, ertapenem, daptomycin, linezolid, and tigecycline with guidelines for use (Table S1). This is on the basis of downstream ecologic, clinical, and health economic impacts with drug-resistant pathogens.^6,7^ There are further risks with drug-associated adverse events, cost, and other factors resulting in this policy decision. There are no health-authority mandated continuing medical education obligations pertaining to infectious diseases (ID) or antimicrobial stewardship for staff providers or medical trainees at the time of this study. All clinicians have free public access to Bugs & Drugs,^8^ the official AHS prescribing resource, and other AHS prescribing tools.

University of Alberta medical students’ primary education regarding antimicrobial stewardship is at the beginning of a four-year undergraduate program where ID, microbiology, antimicrobial pharmacology, and antimicrobial stewardship are taught. Postgraduate medical trainees may have clinical ID rotations depending on individual residency training program requirements stipulated by the Royal College of Physicians and Surgeons of Canada.

With this context, two separate needs assessment identified 28% of the six AHS restricted antimicrobials were prescribed inappropriately in 2014, and 29% in 2017 at the University of Alberta Hospital (UAH), an AHS operated 700-bed tertiary care center in Edmonton, Canada. This informed the UAH to launch an ASP in April 2018 consisting of PAF of these six restricted antibiotics. Hospitalized adults 18 years or older are reviewed in the initiative when prescribed an incident prescription of one of six restricted antibiotics: meropenem, imipenem, ertapenem, daptomycin, linezolid, and tigecycline. Patients are excluded from review if the prescription was discontinued prior to the completion of audit, prescribed for prophylaxis (ie, carbapenem for high-risk patients prior to transrectal prostatic biopsy^8^), or prescribed based on recommendations from the ASP team. Prescriptions are also excluded from PAF if the patient was discharged from hospital prior to audit completion, defined as completion of feedback discussion with the attending physician. Each audit is conducted independently by an ASP pharmacist and ASP physician, then reviewed together to reach consensus assignment of appropriateness and subsequent recommendations if any. ASP pharmacists have either a PGY-2 ID/ASP residency or have completed an extensive locally-developed antimicrobial stewardship training program plus the Society of Infectious Diseases Pharmacists Antimicrobial Stewardship Certificate Program. ASP physicians are Royal College of Physicians and Surgeons of Canada certified ID specialists. Audits are carried out daily on weekdays with weekend prescriptions audited on the following operational day. Comprehensive chart review is conducted and the restricted antibiotic, regimen, and duration are assessed. Appropriateness is evaluated and assigned based on case-specific context, incorporating local antibiogram and the patient’s past microbiology results, and institutional prescribing guidelines^8^ or expert opinion if guidelines were not available. Real time verbal feedback with recommendations to optimize the prescription, if applicable, is provided to the most responsible physician with written feedback contemporaneously recorded in the patient’s chart using a standardized, templated note. Quality improvement (QI) metrics including patient age, prescriber role, attending service, antimicrobial information (dosage, duration), indication, appropriateness, and PAF recommendations are recorded prospectively in the ASP QI database. PAF of restricted antimicrobials is an ongoing longitudinal initiative at UAH since implementation in 2018.

There is little published quantitative data exploring prescriber role and its relationship on antibiotic prescription appropriateness^9,10^ and if prescription timing factors have any impact.^11,12^ Pragmatic research examining this may help ASPs make informed, evidence-based operational decisions to inform PAF implementation while balancing return-on-investment. We therefore aimed to study the relationship of prescriber training level and timing factors with prescribing appropriateness in six restricted antibiotics as audited by ASP PAF.

## Methods

### Study design and objectives

This was a single-center 5-year retrospective cohort study. Our primary objective was to compare prescriber roles (medical trainee vs staff) with restricted antibiotic prescribing appropriateness. Secondary objectives were to assess the impact of select timing factors, such as time of day and day of week, and other independent variables available in the ASP QI database thought to possibly impact prescription appropriateness. Secondary objectives were developed based on literature suggesting that timing factors may impact prescription appropriateness.^10–12^ Assessment of prescribing appropriateness during the COVID-19 pandemic was included based on prior studies suggesting poor antimicrobial stewardship during the pandemic.^14,15^ Appropriate empiric antimicrobials are associated with reduced mortality and was also included.^16^ Other independent variables including prescriber specialty and patient age were included based on possible clinical significance.

### Inclusion and exclusion criteria

All restricted antimicrobials subjected to ASP PAF between April 2018 and March 2023 were identified from our center’s ASP QI database and included. Exclusion criteria were those audits whereby prescriber role or prescription date or time of day were not available.

### Definitions of training level

Medical students, residents, subspecialty residents, and fellows were considered medical trainees and attending physicians or surgeons were considered staff. Those with other independent practitioner roles, including clinical associates, hospitalists, physician assistants, and pharmacists were also considered staff.

### Ethics approval

Ethics approval was granted by the University of Alberta (Pro00140246).

### Data collection and outcomes evaluation

All data, including prescriber role and ASP assigned prescription appropriateness, was extracted from the ASP QI database. The primary outcome was prescription appropriateness comparing medical trainees and staff. Secondary outcome timing factors included time of day (daytime vs after-hours), day of week (weekday vs weekend), academic quarter, and the period relative to the COVID-19 pandemic. Timing relative to the COVID-19 pandemic was included given the significant change in admitted patient populations in our hospitals. Academic quarter was a variable included given the hypothesis that medical trainees would be more experienced as the academic year progressed. Other examined variables included prescriber specialty (Critical Care, Emergency Medicine, ID, Medicine, and Surgery), patient age, antimicrobial class (carbapenem vs non-carbapenem), and empiric versus culture-directed prescriptions. Antimicrobial class was included based on the hypothesis that clinicians may be more familiar with prescribing carbapenems than non-carbapenems.

Daytime working hours were defined as 08:00–17:59 and after-hours were defined as 18:00–07:59. Weekends included Saturday, Sunday, and statutory holidays. The academic year at our institution begins July 1 (eg, quarter 1 is July-September). The COVID-19 pandemic period was defined as March 2020 through May 2022.

Categorical variables were summarized as frequencies and percentages. Continuous variables were summarized as median and interquartile range. A multiple logistic regression analysis of all individual prescriptions was conducted to identify factors associated with appropriate prescriptions using whether a prescription was appropriate as the outcome variable. Variables included were prescriber role, timing factors, and the other examined variables listed above. Values were reported as adjusted odds ratios (aOR) with 95% confidence intervals. Significance was defined with a *P* value less than .05. GraphPad Prism (version 10.2.2 for Windows, GraphPad Software, Boston, Massachusetts USA) was used to perform analyses and create figures.

Given institutional practice where all ID medical trainee prescriptions are reviewed with ID staff prior to suggesting or ordering, a prespecified secondary analysis was completed assigning all ID trainee prescriptions to the staff role. In addition, medical trainee-ordered prescriptions during working hours were assigned to the staff category in this secondary analysis, based on the assumption that these prescriptions were reviewed with or directed by staff during working hours. Medical trainee-ordered prescriptions ordered after-hours were not assigned as staff prescriptions in this secondary analysis as there may have been reduced staff and pharmacy services support after-hours.

## Results

Over the 5-year study period, 3,741 prescriptions underwent PAF; 54 were excluded (20 lacked prescription time and 33 lacked prescriber role data). A total of 3,687 prescriptions were analyzed.

The median patient age was 62 years (interquartile range, 48–71). Overall, 47.2% (1,739/3,687) of all prescriptions were ordered by staff with 68.3% (1,188/1,739) appropriate compared to 68.6% (1,336/1,948) for medical trainees. There were more prescriptions written during working hours (2,464/3,687 or 66.8%) and during weekdays (2,773/3,687 or 75.2%). The most audited restricted antibiotics were carbapenems (3,160/3,687 or 85.7%). Medicine services had the highest volume of prescriptions (1,719/3,687 or 46.6%). Prescription volumes increased over time (Figure S1) but were similar when comparing quarters (Table 1). Baseline prescription characteristics and the percentage of appropriate prescriptions for each variable are shown in Table 1.

**Table 1.** Characteristics and appropriateness of restricted antibiotic prescriptions audited by prospective audit and feedback

Independent variable	Total *N* = 3,687	Appropriate *N* = 2,524	Not appropriate *N* = 1,163
**Prescriber role, *n* (%)**			
Staff	1,739	1,188 (68.3)	551 (31.7)
Medical trainee	1,948	1,336 (68.6)	612 (31.4)
**Time of day, *n* (%)**			
Working hours (08:00 – 17:59)	2,464	1,706 (69.2)	758 (30.8)
Staff	1,313	903 (68.8)	410 (31.2)
Medical trainee	1,151	803 (69.7)	348 (30.2)
After-hours (18:00 – 07:59)	1,223	818 (66.9)	405 (33.1)
Staff	426	285 (66.9)	141 (33.1)
Medical trainee	797	533 (66.9)	264 (33.1)
**Time of week, *n* (%)**			
Weekday (Monday-Friday)	2,773	1,948 (70.2)	825 (29.8)
Weekend (Saturday-Sunday)	914	576 (63.0)	338 (37.0)
**Academic quarter, *n* (%)**			
Q1 (July-September)	1,055	717 (67.9)	338 (32.1)
Q2 (October-December)	730	512 (70.1)	218 (29.9)
Q3 (January-March)	985	659 (66.9)	326 (33.1)
Q4 (April-June)	917	636 (69.4)	281 (30.6)
**Relation to COVID-19 pandemic, *n* (%)**			
Pre-COVID-19	1,056	593 (56.2)	463 (43.8)
COVID-19	1,749	1,252 (71.5)	497 (28.5)
Post-COVID-19	882	679 (77.0)	203 (23.0)
**Prescribing specialty, *n* (%)**			
Critical care	810	596 (73.6)	214 (26.4)
Emergency medicine	52	36 (69.2)	16 (30.8)
Infectious diseases	552	483 (87.5)	69 (12.5)
Medicine	1,719	1,065 (62.0)	654 (38.0)
Surgery	554	344 (62.1)	210 (37.9)
**Median patient age, year (IQR)**	62 (48–71)	64 (51–74)	61 (47 – 70)
**Antimicrobial class, *n* (%)**			
Carbapenem	3,160	2,099 (66.4)	1,061 (33.6)
Meropenem	2,884	1,980 (68.7)	904 (31.3)
Imipenem	111	53 (47.7)	58 (52.3)
Ertapenem	165	66 (40.0)	99 (60.0)
Non-carbapenem	527	425 (80.6)	102 (19.4)
Daptomycin	223	198 (88.8)	25 (11.2)
Linezolid	297	221 (74.4)	76 (25.6)
Tigecycline	7	6 (85.7)	1 (14.3)
**Culture-directed vs empiric, *n* (%)**			
Empiric	2,672	1,899 (71.1)	773 (28.9)
Culture-directed	1,015	625 (61.6)	390 (38.4)

Q, academic quarter; COVID-19, Coronavirus Disease 2019; IQR, interquartile range.

**Figure 1.**
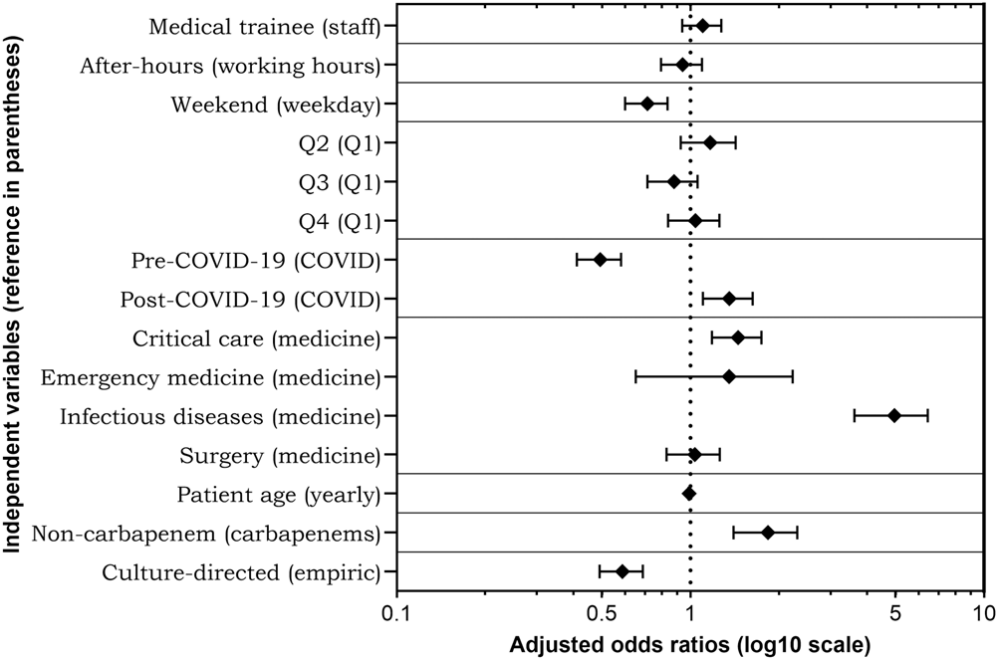
Adjusted odds ratios of each independent variable. The *Y*-axis shows each individual variable with the reference values for discrete variables noted in parentheses. The *X*-axis adjusted odds ratios are represented by a log10 scale with error bars showing the 95% confidence interval. Q, academic quarter; COVID-19, Coronavirus Disease 2019.

**Figure 2.**
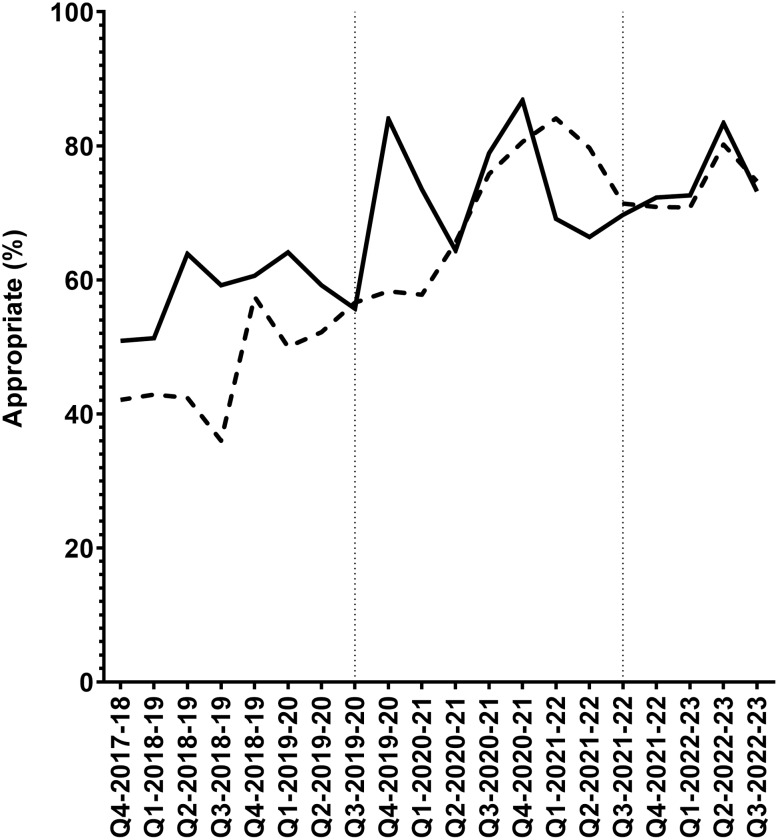
Percent of appropriate restricted antimicrobial prescriptions over time. Solid line represents the percentage of restricted antibiotics ordered during working hours and assessed as appropriately prescribed. The dashed line represents the percentage of restricted antibiotics ordered after-hours and assessed as appropriately prescribed. Vertical dashed lines indicate boundaries of the Coronavirus Disease 2019 pandemic (March 2020 through May 2022). Q, academic quarter.

Of all prescriptions included in the study, 1,163 (31.5%) were assessed by ASP PAF as inappropriate. The aOR for each independent variable with regards to appropriateness are shown in Figure 1 and Table S2. Medical trainee prescribing appropriateness was similar to staff (aOR 1.09 [0.94–1.28], *P* = .25). After-hours prescription appropriateness did not differ (aOR 0.93 [0.79–1.10], *P* = .39) but weekend prescriptions were observed to be significantly less appropriate than weekdays (aOR 0.71 [0.60–0.84], *P* < .0001). Prescribing appropriateness improved over time: from pre-pandemic (prior to March 2020) appropriateness of 56.2% to 71.5% during the COVID-19 pandemic (March 2020 to May 2022) to 76.9% post-pandemic (after May 2022), as shown in Figure 2.

A secondary analysis was conducted assigning prescriptions written by ID trainees and all prescriptions written during working hours, regardless of who prescribed, to the staff category. A total of 2,919/3,687 (79.4%) prescriptions were attributed to staff with 68.9% appropriateness compared to 66.4% in medical trainees, with no difference in the odds of appropriateness (aOR 1.28 [0.98–1.67], *P* = .066). Removing prescriptions where ID was involved in care from the secondary analysis did not significantly affect the odds of appropriateness (aOR 1.11 [0.94–1.30], *P* = .22).

## Discussion

In our study examining appropriateness of restricted antibiotics subject to ASP PAF, we observed inappropriate prescribing of restricted antimicrobials was similar amongst medical trainees and staff; however, inappropriate prescriptions were common on weekends, regardless of prescriber. During implementation of this PAF initiative at our center, concerns were raised that prescribing appropriateness differed amongst staff and medical trainees and that a more focused ASP intervention targeting one of these prescriber groups would yield higher return-on-investment. While there are many reported ASP initiatives targeting medical trainees,^10,13^ there is little comparative literature reporting antibiotic prescribing appropriateness by prescriber role/training level, and the available literature is mostly based on staff experience, rather than role.^14,15^ Our study provides data to inform the future of PAF initiative, not only at our site, but provides evidence to include both medical trainees and staff alike in future interventions. As inappropriate prescribing was common in both groups, the development of future prescribing resources and medical education leveraging a wider array of digestible and acceptable formats may reach a broader audience more effectively.^13,16–18^

Our study also aligns with other studies that the time of prescribing may be associated with antibiotic prescription appropriateness.^11,12,19,20^ We initially hypothesized that given reduced oversight and/or pharmacy services support after-hours and during weekends, medical trainee prescribing would be less appropriate. Indeed, weekend prescriptions were more likely to be inappropriate although this was not the case for after-hours prescriptions. The potential reasons for the lack of difference in appropriateness after-hours include that prescriptions may be deferred to working hours; advice may be sought from other on-site prescribers overnight, or the study may simply be underpowered to detect a difference. Further studies into the timing factors associated with prescribing appropriateness are necessary to draw conclusions to inform implementation of prescribing supports to mitigate these factors.

Over the 5-year study period, prescription appropriateness for restricted antibiotics at our center improved from 56.2% to 71.5% throughout the pandemic and 76.9% post-pandemic. We do not believe this was due to increasing AMR resulting in empiric restricted antimicrobials being more appropriate over time as audits are individualized based on both local antibiograms and case-specific microbiology results. Furthermore, the percentage of antimicrobial resistant *Staphylococcus aureus*, *Enterococcus faecium*, and *Escherichia coli* isolates from clinical specimens collected at UAH over the study period did not drastically change (Table S3 and S4). The educational component of our center’s longitudinal restricted antibiotic PAF initiative, which involves written and verbal recommendations directly to the attending physician, likely contributed the improvement of prescribing appropriateness over time, similar to reports elsewhere.^4^ Finally, the COVID-19 pandemic period was not associated with reduced restricted antibiotic prescribing appropriateness at our center. This may be due to the organization’s commitment to maintain existing stewardship resources and staffing to support the PAF of Restricted Antibiotics Initiative through the pandemic.^21^

This study had several limitations. Our data represents the prescribing practice of a single center; replication of these findings in other centers would be important to inform data-driven ASP initiatives. In our case, our data informed the future direction of our PAF initiative such that it will remain inclusive of medical trainees and staff alike. Our study did not consider the impact of staffing levels, staff years in practice, patient volumes by prescriber, or pharmacy support as these were not available through chart review, but these factors may have an impact on appropriateness and will be important considerations for future study. Other notable limitations are the lack of inclusion of ID syndrome or further analysis of appropriateness by individual agents. This would be difficult to include unless other variables were limited to maintain appropriate numbers of audits for analysis and a higher number of the low-frequency antimicrobials, such as tigecycline, were available. Another limitation is that our analysis did not include short courses of restricted antimicrobials limiting assessment of appropriateness to those restricted antibiotics with a fully completed ASP audit. In our secondary analysis, there was an assumption as to who was authorizing the prescription; during daytime hours, it was assumed that staff were included in decisions to prescribe restricted antimicrobials, but it is not easily determined whether this is always the case. Further, it is unclear how frequently after-hour prescriptions involved undocumented collaboration with staff. Our study revealed a substantial degree of inappropriate prescriptions in all specialties (Table 1) and factors to explore this finding were not determined a-priori and are a limitation of this study. Finally, we were unable to determine and differentiate the level of training within the medical trainee cohort (medical students, residents, subspecialty residents, and fellows) due to the retrospective nature of the study and constant influx and efflux of medical trainees in our citywide hospital-based training system.

In conclusion, medical trainees did not prescribe restricted antibiotics more inappropriately than staff, as assessed by ASP PAF at our center. This data informed the future direction of our ASP initiative to remain inclusive of all medical trainees and staff alike. Implementation of additional resources and supports to improve weekend prescribing appropriateness will be explored. Further studies to examine timing factors and antibiotic prescribing appropriateness are needed.

## Supporting information

10.1017/ash.2025.10185.sm001 Greidanus et al. supplementary material Greidanus et al. supplementary material

## Supplementary material

The supplementary material for this article can be found at https://doi.org/10.1017/ash.2025.10185.

## Research Transparency and Reproducibility

Data available on request.
