# Supplementary material for: The relationship of prescriber training level to restricted antibiotic prescribing appropriateness at a tertiary academic hospital: a retrospective study
Source: Antimicrob Steward Healthc Epidemiol. 2025 Oct 19;5(1):e280. doi: 10.1017/ash.2025.10185 (PMC12538364; doi:10.1017/ash.2025.10185)
Supplement: Greidanus et al. supplementary material [file S2732494X2510185Xsup001.docx]

Supplementary Data

**Table S1. Alberta Health Services Provincial Drug Formulary Restriction Usage Guidelines for the six studied restricted antimicrobials.**

| Antimicrobial | Formulary Restriction Usage Guidelines |
| --- | --- |
| Meropenem | 1. Therapy of severe infections involving Gram negative organisms in patients who are intolerant of, or unresponsive to, or whose isolates are suspected or documented to be resistant (e.g. ESBL, inducible (AmpC) ß-lactamases) to, first line agents and piperacillin-tazobactam  2. Therapy of severe suspected or documented polymicrobial infections in patients who are intolerant of, or unresponsive to, or whose isolates are suspected or documented to be resistant to, first line agents and piperacillin-tazobactam. NB: Some suggest imipenem preferred if Gram positive cocci in chains are predominant on Gram stain and Enterococcus faecalis is a probable pathogen, or Enterococcus faecalis is proven by culture, based on its in vitro activity; however there are no clinical data suggesting differential response.)  3. Empiric therapy of post-traumatic/post-neurosurgical meningitis in combination with vancomycin  4. Alternative to ceftazidime for therapy of central nervous system (CNS) infections due to Pseudomonas aeruginosa  5. Therapy of infections involving multi-drug resistant Pseudomonas aeruginosa where there is documented susceptibility to meropenem  6. Empiric therapy in high risk febrile neutropenic patients (oral temperature greater than/equal to 38.3°C once or greater than/equal to 38°C for greater than/equal to 1 hour, absolute neutrophil count less than 0.5 x 10exp9/L) +/- aminoglycoside |
| Imipenem- cilastatin | 1. Therapy of severe infections involving Gram negative organisms in patients who are intolerant of, or unresponsive to, or whose isolates are suspected or documented to be resistant (e.g. ESBL, inducible (AmpC) ß-lactamases) to, first line agents and piperacillin-tazobactam (meropenem preferred as less expensive than imipenem)  2. Therapy of severe suspected or documented polymicrobial infections in patients who are intolerant of, or unresponsive to, or whose isolates are suspected or documented to be resistant to, first line agents and piperacillin-tazobactam (meropenem preferred as less expensive than imipenem. Some suggest imipenem preferred if Gram positive cocci in chains are predominant on Gram stain and Enterococcus faecalis is a probable pathogen, or Enterococcus faecalis is proven by culture, based on its in vitro activity; however there are no clinical data suggesting differential response.)  3. As part of combination therapy of infections with Nocardia spp or nontuberculous Mycobacteria spp  4. Empiric therapy in high risk febrile neutropenic patients (oral temperature greater than/equal to 38.3°C once or greater than/equal to 38°C for greater than/equal to 1 hour, absolute neutrophil count less than 0.5 x 10exp9/L) +/- aminoglycoside (meropenem preferred as less expensive than imipenem) |
| Ertapenem | 1. Empiric therapy of polymicrobial complicated skin and skin structure infections, including bite wound infections  2. Therapy of infections due to Enterobacteriaceae producing inducible (AmpC) ß-lactamases or extended-spectrum ß-lactamases (ESBLs) where there is resistance to first line agents and documented susceptibility to ertapenem  3. Empiric therapy for patients at high risk (e.g. previous ESBL infection, international travel history) of infections due to Enterobacteriaceae producing extended-spectrum ß-lactamases (ESBLs)  4. Therapy of community-acquired intra-abdominal infections in patients intolerant or unresponsive to first line therapy (ceftriaxone + metronidazole) |
| Daptomycin | Therapy of serious infections (excluding those of the lower respiratory tract), such as complicated skin & skin structure infections, bacteremia and infectious endocarditis:  1. Due to Gram positive bacteria (e.g. methicillin resistant S. aureus (MRSA), methicillin resistant coagulase negative Staph (MRCoNS), vancomycin resistant Enterococci (VRE))AND  2. Used in those patients who are allergic to, or who are intolerant of, or whose infections are refractory to, or whose isolates have reduced susceptibility or resistance to, alternative formulary agents, such as vancomycin, or linezolid. |
| Linezolid | 1. Therapy of vancomycin resistant Enterococcus (VRE) infections where no other formulary alternatives are suitable.  2. Therapy of methicillin resistant S. aureus (MRSA) pneumonia  3. Therapy of other methicillin resistant S. aureus (MRSA), and methicillin resistant coagulase negative Staph (MRCoNS) infections in patients who are allergic to, or who are intolerant of, or whose infections are refractory to, or whose isolates have reduced susceptibility or resistance to, vancomycin  4. Therapy of susceptible organisms in patients who are allergic to, or who are intolerant of, or whose infections are refractory to, or whose isolates have reduced susceptibility or resistance to all other formulary alternatives (e.g. ß-lactams, vancomycin) |
| Tigecycline | 1. Therapy of infections (other than UTIs, meningitis, febrile neutropenia, and pseudomonal) involving multi-drug resistant organisms (e,g. MRSA, VRE, ESBL) in patients who are refractory to, allergic to, intolerant of, or whose pathogens are resistant to, all alternative formulary agents (e.g. MRSA - vancomycin, linezolid; VRE - linezolid; ESBL - imipenem, meropenem, aminoglycosides, quinolones, TMP/SMX, etc.) and where there is documented susceptibility to tigecycline  2. Therapy of polymicrobial infections in patients who are refractory to, allergic to, intolerant of, or whose pathogens are resistant to, first line agents and where there is documented susceptibility to tigecycline  NB: Use tigecycline with caution in patients with suspected/documented bacteremia (e.g. clinical history of rigors/positive blood cultures) due to its low serum concentrations. |

**Table S2: Adjusted odds ratio estimates with 95% confidence interval (CI).**

| **Variable** | **Adjusted odds ratio** | **95% Confidence interval** | **P value** |
| --- | --- | --- | --- |
| Medical trainee (Staff) | 1.094 | 0.9379 to 1.275 | 0.2534 |
| After-hours (Working hours) | 0.9324 | 0.7945 to 1.095 | 0.3923 |
| Weekend (Weekday) | 0.7072 | 0.5988 to 0.8359 | <0.0001 |
| Q2 (Q1) | 1.149 | 0.9266 to 1.426 | 0.2069 |
| Q3 (Q1) | 0.869 | 0.7138 to 1.058 | 0.1617 |
| Q4 (Q1) | 1.027 | 0.8398 to 1.256 | 0.795 |
| Pre-COVID-19 (COVID-19) | 0.4881 | 0.4104 to 0.5802 | <0.0001 |
| Post-COVID-19 (COVID-19) | 1.339 | 1.103 to 1.629 | 0.0034 |
| Critical care (Medicine) | 1.021 | 0.8293 to 1.258 | 0.8477 |
| Emergency medicine (Medicine) | 1.435 | 1.183 to 1.745 | 0.0003 |
| Infectious diseases (Medicine) | 4.795 | 3.621 to 6.431 | <0.0001 |
| Surgery (Medicine) | 1.181 | 0.6517 to 2.231 | 0.5943 |
| Patient age (Yearly) | 0.9908 | 0.9864 to 0.9951 | <0.0001 |
| Non-carbapenem (Carbapenems) | 1.794 | 1.402 to 2.312 | <0.0001 |
| Culture-directed (Empiric) | 0.5814 | 0.4909 to 0.6886 | <0.0001 |

Area under the receiver operating characteristic curve was 0.6901, p<0.0001. Log-likelihood ratio (G^2^) was 379.8 rejecting the null hypothesis that a simpler intercept only model is correct (p<0.0001). Q: academic quarter; COVID-19: Coronavirus Disease 2019.

**Table S3. The percentage of methicillin-susceptible *Staphylococcus aureus* and vancomycin-susceptible *Enterococcus faecium* isolates from clinical specimens collected at the University of Alberta Hospital from 2018 to 2023.**

| Year | *Staphylococcus aureus* isolates from clinical specimens with methicillin susceptibility testing (n) | *Staphylococcus aureus* isolates susceptible to methicillin (%) | *Enterococcus faecium* isolates from clinical specimens with vancomycin susceptibility testing (n) | *Enterococcus faecium* isolates susceptible to vancomycin (%) |
| --- | --- | --- | --- | --- |
| 2018 | 2035 | 77 | 323 | 71 |
| 2019 | 2021 | 77 | 338 | 78 |
| 2020 | 1600 | 76 | 284 | 73 |
| 2021 | 1784 | 76 | 304 | 64 |
| 2022 | 1625 | 75 | 314 | 61 |
| 2023 | 2760 | 71 | 480 | 68 |

**Table S4. The percentage of ceftriaxone and piperacillin-tazobactam susceptible *Escherichia coli* isolates from clinical specimens collected at the University of Alberta Hospital from 2018 to 2023.**

| Year | *Escherichia coli* isolates from clinical specimens with ceftriaxone and/or piperacillin-tazobactam susceptibility testing (n) | *Escherichia coli* isolates susceptible to ceftriaxone (%) | *Escherichia coli* isolates susceptible to piperacillin-tazobactam (%) |
| --- | --- | --- | --- |
| 2018 | 1768 | 86 | 83 |
| 2019 | 1704 | 86 | 89 |
| 2020 | 1591 | 86 | 95 |
| 2021 | 1727 | 86 | 97 |
| 2022 | 1602 | 85 | 96 |
| 2023 | 2510 | 79 | 98* |

* A total of 2445 E. coli isolates received piperacillin-tazobactam susceptibility testing in 2023.

**Figure S1. Volume of restricted antimicrobial prescriptions audited over time.**

Q: Academic quarter
